# Crystal structure of μ_6_-chlorido-nona­kis­(μ-4-chloro­pyrazolato)bis-μ_3_-methoxo-hexa­copper(II)

**DOI:** 10.1107/S2056989017001189

**Published:** 2017-01-27

**Authors:** Kaige Shi, Logesh Mathivathanan, Raphael G. Raptis

**Affiliations:** a Department of Chemistry and Biochemistry, Florida International University, 11200 SW 8th Street, Miami, FL 33199, USA

**Keywords:** crystal structure, trigonal prismatic hexa­nuclear copper pyrazolate, μ_6_-Cl coordination, chloride encapsulation

## Abstract

The hexa­nuclear title compound, [{Cu_3_(μ_3_-OCH_3_)(μ-C_3_H_2_ClN_2_)_3_}_2_(μ-C_3_H_2_ClN_2_)_3_(μ_6_-Cl)] or [Cu_6_(C_3_H_2_ClN_2_)_9_(CH_3_O)_2_Cl], crystallizes in the space group *Pbcn*, with individual mol­ecules being located on a twofold rotation axis. The mol­ecule adopts a trigonal prismatic shape, with two trinuclear units linked by three 4-chloro­pyrazolate ligand bridges by encapsulating a Cl^−^ anion in a μ_6_-coordination mode. In the crystal, individual mol­ecules are stacked into rods parallel to [1-10] that are arranged in a pseudo-hexa­gonal packing. Cohesion between mol­ecules is accomplished through weak C—H⋯Cl inter­actions.

## Abstract 2

The hexa­nuclear copper pyrazolato complex has a trigonal prismatic shape and contains an encapsulated chloride ligand.

## Chemical context   

Multinuclear transition metal ion complexes often have inter­esting properties, such as magnetic, electrochemical, and catalytic functions. *N*-donor ligands have coordination plast­icity and large affinity for transition metals, and their employment has provided structures of various nuclearities and dimensionalities, which have been shown to be of inter­est in catalysis, bio-inorganic chemistry and mol­ecular magnetism. There have been several reports concerning multinuclear copper(II) complexes supported by pyrazolato (pz^−^) bridging ligands. In this context, we have investigated a family of redox-active Cu_6_-pyrazolato complexes with trigonal prismatic shapes (Mezei *et al.*, 2007 ▸; Zueva *et al.*, 2009 ▸), including one with a μ_6_-F central ligand (Mathivathanan *et al.*, 2015 ▸). In connection with our earlier work, the title compound, [{Cu_3_(μ_3_-OCH_3_)(μ-C_3_H_2_N_2_Cl)_3_}_2_((μ-C_3_H_2_N_2_Cl)_3_(μ_6_-Cl)], has been prepared recently; it contains an encapsulated μ_6_-Cl ligand at the center of the hexa­nuclear complex.
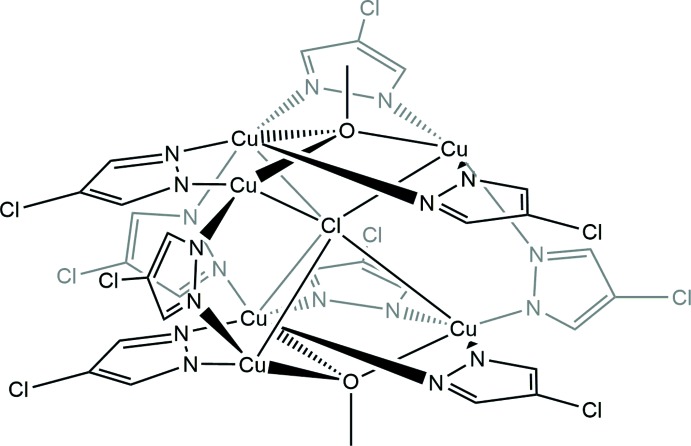


## Structural commentary   

**Figure 1:**
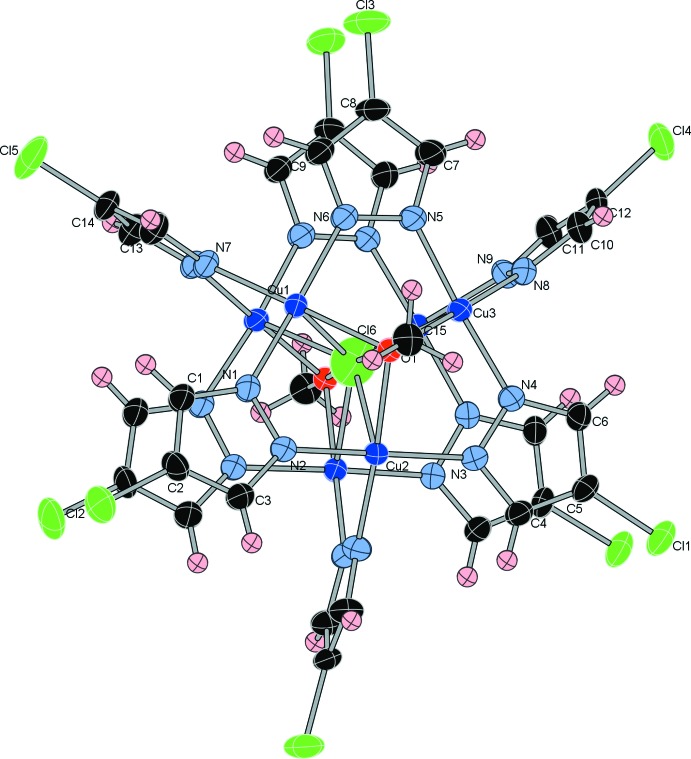
The mol­ecular structure of the title compound, showing the atom-labeling scheme. H atoms are not shown for clarity. Displacement ellipsoids are drawn at the 40% probability level. Non-labeled atoms are related to the labeled atoms by the symmetry operation (−*x*, *y*, −*z* + 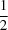).

The crystal structure of the title compound (Fig. 1 ▸) consists of two trinuclear [Cu_3_(μ_3_-OMe)(μ-4-Cl-pz)_3_]^2+^ (OMe is a methoxide, 4-Cl-pz a 4-chloro­pyrazolato ligand) units bridged by three μ-4-Cl-pz^−^ ligands; the complete mol­ecule adopts .2. point group symmetry. The six Cu^II^ ions form a trigonal prismatic array and a chloride ion is located at the center of the cage, coordinating to the two {Cu}_3_ units in a μ_6_ mode. All six Cu^II^ atoms are five-coordinate with distorted square-pyramidal N_3_OCl coordination sets with the Cl atom occupying the apical position. Each Cu_3_ triangle is capped by an OMe group with the O atom 0.8472 (1) Å above the Cu_3_ plane, a somewhat smaller deviation from the Cu_3_ plane than the one found in the previously reported structure of [{Cu_3_(μ_3_-OMe)(μ-pz)_3_}_2_(μ-pz)_3_(μ_6_-Cl)], where μ_3_-bridging meth­oxy groups are located *ca* 1.0 Å above this plane (Kamiyama *et al.*, 2002 ▸). The distance between two Cu_3_ planes is 3.3858 (2) Å. The six Cu—O bond lengths range from 2.033 (2)–2.044 (2) Å, while the Cu—O—Cu angles are in the 102.70 (10)–105.62 (10)° range. The Cu⋯Cu distances within each triangle, 3.1801 (9)–3.2526 (9) Å, are slightly shorter than those between the triangles, 3.356 (2)–3.401 (2) Å). The μ_6_-Cl ligand is located close to the center of the trigonal prism defined by the six Cu atoms and non-equidistant from the three pairs of Cu^II^ ions. The longest Cu—Cl distance of 2.6222 (13) Å (Cu2) is longer than the sum of the ionic radii (2.38 Å), indicating that the two [Cu_3_(μ_3_-OMe)(μ-4-Cl-pz)_3_]^2+^ units are not templated by the encapsulated chloride. The other two Cu—Cl bond lengths are 2.424 (2) (Cu1) and 2.4859 (13) Å.

Differences in structural parameters between the four known {Cu_6_-pyrazolato} complexes with trigonal prismatic shape are compiled in Table 1 ▸. The inter-trimer and intra-trimer Cu⋯Cu distances are shorter in the title compound than those in the [Cu_6_Cl] compound reported earlier with 4-H-pz as a ligand (Kamiyama *et al.*, 2002 ▸), indicating the effect of electron-withdrawing Cl-substitution of the pyrazolato ligands. The Cu—N distances of the pyrazolato ligands connecting the two trimers are longer compared to those in {Cu_6_-μ_6_-F} (Mathivathanan *et al.*, 2015 ▸) or {Cu_6_-μ_6_-Cl} (Kamiyama *et al.*, 2002 ▸). However, the Cu—N distances are similar to those in the empty Cu_6_-pyrazolato cage (Mezei *et al.*, 2007 ▸).

## Supra­molecular features   

In the trigonal prismatic mol­ecules, the six pyrazolato ligands of the eclipsed {Cu_3_-pyrazolato} trimers exhibit weak π–π stacking inter­actions, with centroid-to-centroid distances of 3.8489 (6) and 3.6059 (6) Å. These distances are comparable to the ones found in the Cu_6_-pyrazolato complex with no encapsulated anion, where the pyrazolato ring centroids are 3.741 (6), 3.700 (6) and 3.680 (6) Å apart (Mezei *et al.*, 2007 ▸).

**Figure 2:**
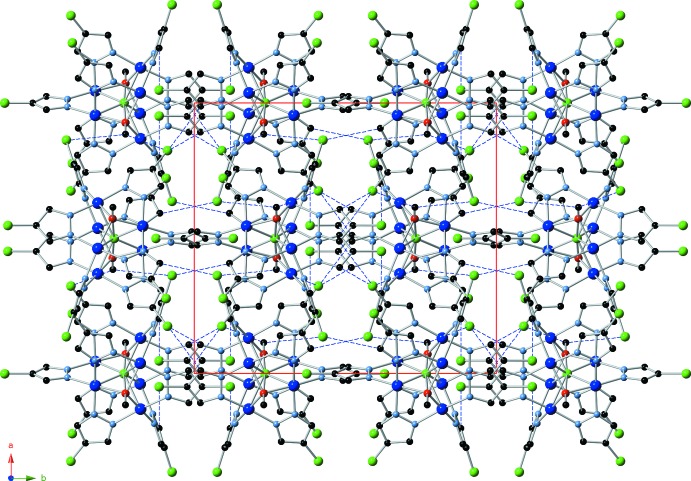
Crystal packing diagram viewed along [001], showing hydrogen bonds as blue dashed lines.

**Figure 3:**
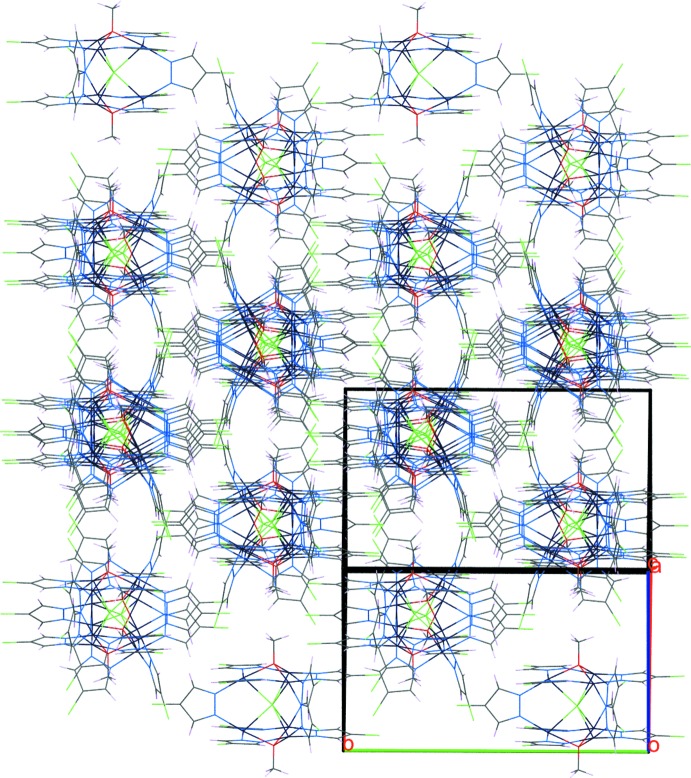
Crystal packing diagram viewed along [1
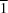
0], highlighting the pseudo-hexa­gonal rod packing of {Cu_6_} mol­ecules.

While conventional hydrogen bonds are absent in the structure, there are three weak inter­molecular C—H⋯Cl inter­actions observed in the crystal structure (Fig. 2 ▸ and Table 2 ▸). Individual {Cu_6_-μ_6_ *-*Cl}-mol­ecules are stacked in rods parallel to [1
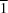
0] that, in turn, are arranged in a pseudo-hexa­gonal packing (Fig. 3 ▸).

## Database survey   

Polynuclear complexes with a μ_6_-coordinating halide anion are not uncommon. However, they are rarely encountered in a trigonal prismatic environment. According to the Cambridge Structure Database (Groom *et al.*, 2016 ▸), only three hexa­nuclear Cu_6_-cages with a μ_6_-coordinating halide anion have been reported in the literature: [{Cu_3_(μ_3_-OMe)(μ-pz)_3_}_2_(μ-pz)_3_(μ_6_-Cl)] (pz = pyrazole; Kamiyama *et al.*, 2002 ▸), [{Cu_3_(μ_3_-OMe)(μ-3,5-Me_2_pz)_3_}_2_(μ_6_-F)(μ_2_-OH)] (3,5-Me_2_pz^−^ =3,5-di­methyl­pyrazolato; Cañon-Mancisidor *et al.*, 2014 ▸) and [{Cu_3_(μ_3_-OH)(μ-pz)_3_}_2_(μ-3,5-Ph_2_pz)_3_(μ_6_-F)] (Mathivathanan *et al.*, 2015 ▸).

## Synthesis and crystallization   

The complex was formed in an one-pot reaction when CuCl_2_·2H_2_O (0.06 mmol, 10.2 mg), 4-Cl-pzH (0.09 mmol, 8.9 mg) and ethyl­amine (0.08 mmol, 11.3 µl) were stirred in 10 ml CH_2_Cl_2_ for 24 h at ambient temperature. The green solution was transferred to a test tube after filtration. A 4 ml 1:1 mixture of CH_2_Cl_2_:MeOH (*v*/*v*) was layered over the CH_2_Cl_2_ layer, 1,2-di(4-pyrid­yl)ethyl­ene (1,2-bpe) (0.01mmol, 1.9 mg) in 4 ml MeOH was added as the third layer on top of the lower two. Suitable crystals for X-ray diffraction were obtained one week later. Yield: 29%. Analysis calculated/found for C_29_H_24_Cl_10_Cu_6_N_18_O_2_: C, 25.15/25.22; H,1.75/1.76; N, 18.22/18.17.

## Refinement   

Crystal data, data collection and structure refinement details are summarized in Table 3 ▸. Hydrogen atoms were placed in geometrically calculated positions and refined with a riding model. Structure refinement indicates a minimum (−1.56 e Å^−3^) near the μ_6_-Cl atom (Cl6), which decreases if the structure is refined with a free site-occupation factor for this atom. This can be explained if some of the Cu_6_-cages (< 10%) are vacant. Such a discrepancy is within the experimental error of the CHN elemental analysis, and we decided to refine the model with full occupancy for this Cl atom. In the final cycles, restraints were applied to obtain acceptable *U_ij_* parameters for Cl6.

## Supplementary Material

Crystal structure: contains datablock(s) I. DOI: 10.1107/S2056989017001189/wm5353sup1.cif

Structure factors: contains datablock(s) I. DOI: 10.1107/S2056989017001189/wm5353Isup2.hkl

Supporting information file. DOI: 10.1107/S2056989017001189/wm5353Isup3.cdx

CCDC reference: 1529271

Additional supporting information:  crystallographic information; 3D view; checkCIF report

## Figures and Tables

**Table 1:** Comparison of selected structural parameters (Å)

	{Cu_6_}, PPN*^*a*^*	{Cu_6_Cl}*^*b*^*	{Cu_6_Cl}*^*c*^*	{Cu_6_F}*^*d*^*
Cu⋯Cu (inter-trimer)	2.975 (3), 2.999, 3.028 (3)	3.3557 (10)–3.4005 (10)	3.621 (1), 3.675 (1)	3.281 (2), 3.335 (2), 3.289 (2)
Cu⋯Cu (intra-trimer)	3.206 (4)–3.279 (5)	3.1801 (9)–3.2526 (9)	3.209 (1), 3.233 (1)	3.234 (2)–3.289 (2)
Cu⋯*X*	–	2.424 (2), 2.4858 (14), 2.6221 (13) (*X* = Cl)	2.604 (1), 2.623 (2) (*X* = Cl)	2.383 (5)–2.605 (5) (*X* = F)
Cu⋯(μ_3_-O*R*)	1.883 (1)–1.894 (5)	2.003 (2)–2.044 (2)	2.083 (4), 2.085 (6) (*R* = Me)	2.048 (3)–2.096 (5) (*R* = H)
Cu—N (inter-trimer)	2.003 (7)–2.056 (6)	2.003 (3)–2.004 (3)	1.990 (5)–1.992 (7)	2.018 (6)–2.047 (6)
Cu—N (intra-trimer)	1.934 (7)–1.964 (7)	1.923 (3)–1.954 (3)	1.931 (5)–1.941 (5)	1.942 (5)–1.979 (6)

Notes: (*a*) Mezei *et al.* (2007 ▸); (*b*) this work; (*c*) Kamiyama *et al.* (2002 ▸); (*d*) Mathivathanan *et al.* (2015 ▸).

**Table 2:** Hydrogen-bond geometry (Å, °)

*D*—H⋯*A*	*D*—H	H⋯*A*	*D*⋯*A*	*D*—H⋯*A*
C1—H1⋯Cl4^i^	0.93	2.75	3.586 (4)	149
C6—H6⋯Cl3^ii^	0.93	2.81	3.466 (4)	129
C15—H15*A*⋯Cl3^iii^	0.96	2.82	3.651 (4)	146

Symmetry codes: (i) 
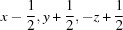
; (ii) 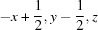; (iii) 
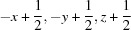
.

**Table 3:** Experimental details

Crystal data	
Chemical formula	[Cu_6_(C_3_H_2_ClN_2_)_9_(CH_3_O)_2_Cl]
*M*_r_	1392.40
Crystal system, space group	Orthorhombic, *P* *b* *c* *n*
Temperature (K)	299
*a*, *b*, *c* (Å)	16.565 (3), 18.474 (4), 14.606 (3)
*V* (Å^3^)	4470.1 (15)
*Z*	4
Radiation type	Mo *K*α
μ (mm^−1^)	3.46
Crystal size (mm)	0.21 × 0.20 × 0.16

Data collection	
Diffractometer	Bruker D8 Quest CMOS
Absorption correction	Multi-scan (*SADABS*; Bruker, 2015 ▸)
*T*_min_, *T*_max_	0.671, 0.745
No. of measured, independent and observed [*I* > 2σ(*I*)] reflections	63202, 5726, 4647
*R*_int_	0.026
(sin θ/λ)_max_ (Å^−1^)	0.674

Refinement	
*R*[*F* ^2^ > 2σ(*F* ^2^)], *wR*(*F* ^2^), *S*	0.041, 0.119, 1.08
No. of reflections	5726
No. of parameters	296
H-atom treatment	H-atom parameters constrained
Δρ_max_, Δρ_min_ (e Å^−3^)	0.75, −1.59

Computer programs: *APEX3* and *SAINT* (Bruker, 2015 ▸), *SHELXT* (Sheldrick, 2015*a* ▸), *SHELXL2016* (Sheldrick, 2015*b* ▸), *OLEX2* (Dolomanov *et al.*, 2009 ▸) and *Mercury* (Macrae *et al.*, 2008 ▸).

## supplementary crystallographic information

### Crystal data

**Table:**

[Cu_6_(C_3_H_2_ClN_2_)_9_(CH_3_O)_2_Cl]	*D*_x_ = 2.069 Mg m^−^^3^
*M**_r_* = 1392.40	Mo *K*α radiation, λ = 0.71073 Å
Orthorhombic, *P**b**c**n*	Cell parameters from 9133 reflections
*a* = 16.565 (3) Å	θ = 3.0–28.6°
*b* = 18.474 (4) Å	µ = 3.46 mm^−^^1^
*c* = 14.606 (3) Å	*T* = 299 K
*V* = 4470.1 (15) Å^3^	Cuboctahedron, green
*Z* = 4	0.21 × 0.20 × 0.16 mm
*F*(000) = 2736	

### Data collection

**Table:**

Bruker D8 Quest CMOS diffractometer	4647 reflections with *I* > 2σ(*I*)
φ and ω scans	*R*_int_ = 0.026
Absorption correction: multi-scan (*SADABS*; Bruker, 2015)	θ_max_ = 28.6°, θ_min_ = 2.9°
*T*_min_ = 0.671, *T*_max_ = 0.745	*h* = −22→22
63202 measured reflections	*k* = −24→24
5726 independent reflections	*l* = −19→19

### Refinement

**Table:**

Refinement on *F*^2^	0 restraints
Least-squares matrix: full	Hydrogen site location: inferred from neighbouring sites
*R*[*F*^2^ > 2σ(*F*^2^)] = 0.041	H-atom parameters constrained
*wR*(*F*^2^) = 0.119	*w* = 1/[σ^2^(*F*_o_^2^) + (0.052*P*)^2^ + 10.494*P*] where *P* = (*F*_o_^2^ + 2*F*_c_^2^)/3
*S* = 1.08	(Δ/σ)_max_ < 0.001
5726 reflections	Δρ_max_ = 0.75 e Å^−^^3^
296 parameters	Δρ_min_ = −1.59 e Å^−^^3^

### Special details

**Table:**

Geometry. All esds (except the esd in the dihedral angle between two l.s. planes) are estimated using the full covariance matrix. The cell esds are taken into account individually in the estimation of esds in distances, angles and torsion angles; correlations between esds in cell parameters are only used when they are defined by crystal symmetry. An approximate (isotropic) treatment of cell esds is used for estimating esds involving l.s. planes.

### Fractional atomic coordinates and isotropic or equivalent isotropic displacement parameters (Å^2^)

**Table:**

	*x*	*y*	*z*	*U*_iso_*/*U*_eq_	
Cu1	0.04492 (3)	0.32824 (2)	0.35296 (3)	0.03365 (12)	
Cu2	−0.03799 (2)	0.17979 (2)	0.41047 (3)	0.03405 (12)	
Cu3	0.13100 (3)	0.17948 (2)	0.29708 (3)	0.03369 (12)	
O1	0.07222 (13)	0.22859 (12)	0.40330 (15)	0.0298 (5)	
Cl1	0.04787 (7)	−0.11802 (5)	0.36907 (8)	0.0538 (3)	
Cl4	0.36310 (7)	0.07485 (7)	0.03389 (9)	0.0601 (3)	
Cl2	−0.22199 (7)	0.40543 (7)	0.58300 (9)	0.0641 (3)	
Cl3	0.32409 (9)	0.41018 (7)	0.15094 (10)	0.0758 (4)	
Cl5	0.000000	0.62857 (8)	0.250000	0.0824 (6)	
Cl6	0.000000	0.23354 (16)	0.250000	0.1040 (8)	
C5	0.0483 (2)	−0.02467 (18)	0.3635 (2)	0.0358 (7)	
N3	0.01229 (17)	0.08926 (15)	0.3792 (2)	0.0345 (6)	
N4	0.08520 (17)	0.08802 (15)	0.3377 (2)	0.0349 (6)	
N1	−0.04891 (18)	0.33017 (16)	0.4322 (2)	0.0370 (6)	
N5	0.1870 (2)	0.27052 (16)	0.2727 (2)	0.0441 (8)	
C2	−0.1503 (2)	0.3569 (2)	0.5233 (3)	0.0428 (8)	
N7	0.0146 (2)	0.42174 (15)	0.2927 (2)	0.0382 (7)	
C12	0.2735 (2)	0.10042 (19)	0.0850 (3)	0.0383 (8)	
C4	−0.0109 (2)	0.02073 (18)	0.3944 (3)	0.0382 (8)	
H4	−0.059153	0.006471	0.421373	0.046*	
N8	0.18357 (18)	0.13431 (16)	0.1875 (2)	0.0362 (6)	
C1	−0.0924 (2)	0.3849 (2)	0.4661 (3)	0.0447 (9)	
H1	−0.084759	0.433718	0.453080	0.054*	
N6	0.1507 (2)	0.33235 (16)	0.2976 (2)	0.0419 (7)	
C14	0.000000	0.5358 (3)	0.250000	0.0471 (13)	
C8	0.2558 (3)	0.3612 (2)	0.2127 (3)	0.0469 (9)	
C6	0.1075 (2)	0.01918 (19)	0.3278 (3)	0.0399 (8)	
H6	0.155241	0.003367	0.300986	0.048*	
N2	−0.07882 (19)	0.26795 (16)	0.4663 (2)	0.0415 (7)	
C11	0.2026 (2)	0.1179 (2)	0.0414 (3)	0.0439 (9)	
H11	0.193686	0.115749	−0.021388	0.053*	
C3	−0.1403 (2)	0.2838 (2)	0.5216 (3)	0.0515 (10)	
H3	−0.171220	0.250424	0.553841	0.062*	
C9	0.1920 (3)	0.3877 (2)	0.2616 (3)	0.0504 (10)	
H9	0.179311	0.436415	0.268681	0.060*	
C10	0.2602 (2)	0.1112 (2)	0.1762 (3)	0.0427 (8)	
H10	0.297665	0.103841	0.222717	0.051*	
C13	0.0244 (3)	0.4907 (2)	0.3198 (3)	0.0478 (9)	
H13	0.044351	0.505543	0.376243	0.057*	
C7	0.2509 (3)	0.2874 (2)	0.2207 (3)	0.0587 (12)	
H7	0.286269	0.254339	0.194252	0.070*	
N9	0.14862 (17)	0.13828 (17)	0.1038 (2)	0.0385 (7)	
C15	0.1151 (3)	0.2292 (2)	0.4877 (3)	0.0468 (9)	
H15A	0.125602	0.180297	0.506599	0.070*	
H15B	0.083328	0.253186	0.533459	0.070*	
H15C	0.165302	0.254366	0.479851	0.070*	

### Atomic displacement parameters (Å^2^)

**Table:**

	*U*^11^	*U*^22^	*U*^33^	*U*^12^	*U*^13^	*U*^23^
Cu1	0.0352 (2)	0.0261 (2)	0.0396 (2)	0.00276 (15)	0.00961 (17)	0.00267 (16)
Cu2	0.0268 (2)	0.0284 (2)	0.0470 (3)	−0.00146 (15)	0.00342 (17)	−0.00153 (17)
Cu3	0.0340 (2)	0.0270 (2)	0.0401 (2)	−0.00140 (15)	0.00890 (17)	−0.00296 (16)
O1	0.0276 (10)	0.0284 (11)	0.0336 (12)	0.0001 (9)	−0.0001 (9)	−0.0004 (9)
Cl1	0.0662 (7)	0.0266 (4)	0.0688 (7)	−0.0019 (4)	−0.0117 (5)	0.0007 (4)
Cl4	0.0496 (6)	0.0620 (7)	0.0687 (7)	0.0247 (5)	0.0180 (5)	0.0044 (5)
Cl2	0.0610 (7)	0.0633 (7)	0.0681 (7)	0.0130 (5)	0.0296 (6)	−0.0109 (6)
Cl3	0.0879 (9)	0.0632 (7)	0.0764 (8)	−0.0382 (7)	0.0455 (7)	−0.0182 (6)
Cl5	0.1185 (17)	0.0253 (7)	0.1034 (15)	0.000	0.0028 (13)	0.000
Cl6	0.113 (2)	0.1012 (18)	0.0981 (17)	0.000	−0.0218 (16)	0.000
C5	0.0441 (19)	0.0259 (15)	0.0375 (18)	−0.0039 (13)	−0.0094 (15)	−0.0016 (13)
N3	0.0322 (14)	0.0303 (13)	0.0410 (16)	−0.0029 (11)	0.0011 (12)	0.0011 (12)
N4	0.0333 (14)	0.0302 (14)	0.0414 (16)	−0.0020 (11)	0.0024 (12)	−0.0034 (12)
N1	0.0376 (15)	0.0319 (14)	0.0415 (16)	0.0044 (11)	0.0089 (13)	0.0007 (12)
N5	0.0413 (16)	0.0302 (14)	0.061 (2)	−0.0016 (13)	0.0199 (15)	−0.0056 (14)
C2	0.0399 (19)	0.045 (2)	0.043 (2)	0.0057 (16)	0.0091 (16)	−0.0078 (16)
N7	0.0451 (17)	0.0255 (13)	0.0439 (16)	0.0009 (12)	0.0064 (14)	0.0003 (12)
C12	0.0344 (17)	0.0342 (17)	0.046 (2)	0.0086 (14)	0.0083 (15)	−0.0036 (15)
C4	0.0390 (18)	0.0315 (17)	0.044 (2)	−0.0054 (14)	−0.0022 (15)	0.0031 (14)
N8	0.0358 (15)	0.0314 (14)	0.0415 (16)	0.0033 (12)	0.0056 (13)	−0.0024 (12)
C1	0.048 (2)	0.0345 (18)	0.051 (2)	0.0058 (16)	0.0153 (18)	−0.0011 (16)
N6	0.0426 (17)	0.0305 (15)	0.0526 (19)	−0.0034 (12)	0.0162 (15)	−0.0010 (13)
C14	0.057 (3)	0.023 (2)	0.061 (4)	0.000	0.008 (3)	0.000
C8	0.049 (2)	0.042 (2)	0.049 (2)	−0.0180 (17)	0.0173 (18)	−0.0066 (17)
C6	0.0395 (18)	0.0318 (17)	0.048 (2)	0.0013 (14)	0.0001 (16)	−0.0071 (15)
N2	0.0365 (15)	0.0312 (14)	0.0569 (19)	0.0008 (12)	0.0148 (14)	−0.0003 (13)
C11	0.0415 (19)	0.049 (2)	0.0410 (19)	0.0118 (16)	0.0019 (16)	−0.0047 (17)
C3	0.043 (2)	0.043 (2)	0.068 (3)	−0.0017 (17)	0.024 (2)	0.0012 (19)
C9	0.053 (2)	0.0336 (18)	0.065 (3)	−0.0082 (17)	0.020 (2)	−0.0008 (18)
C10	0.0385 (19)	0.043 (2)	0.046 (2)	0.0094 (15)	−0.0017 (16)	−0.0017 (16)
C13	0.061 (2)	0.0336 (19)	0.049 (2)	−0.0001 (17)	0.001 (2)	−0.0037 (16)
C7	0.055 (2)	0.042 (2)	0.079 (3)	−0.0071 (19)	0.036 (2)	−0.012 (2)
N9	0.0304 (14)	0.0407 (16)	0.0444 (17)	0.0036 (12)	0.0013 (12)	−0.0019 (13)
C15	0.050 (2)	0.049 (2)	0.041 (2)	0.0018 (17)	−0.0091 (17)	−0.0009 (17)

### Geometric parameters (Å, º)

**Table:**

Cu1—O1	2.033 (2)	N5—C7	1.340 (5)
Cu1—Cl6	2.424 (2)	C2—C1	1.373 (5)
Cu1—N1	1.938 (3)	C2—C3	1.361 (6)
Cu1—N7	2.003 (3)	N7—N7^i^	1.337 (6)
Cu1—N6	1.932 (3)	N7—C13	1.344 (5)
Cu2—O1	2.039 (2)	C12—C11	1.375 (5)
Cu2—Cl6	2.6222 (13)	C12—C10	1.365 (6)
Cu2—N3	1.923 (3)	C4—H4	0.9300
Cu2—N2	1.943 (3)	N8—C10	1.350 (5)
Cu2—N9^i^	1.998 (3)	N8—N9	1.354 (4)
Cu3—O1	2.044 (2)	C1—H1	0.9300
Cu3—Cl6	2.4859 (13)	N6—C9	1.338 (5)
Cu3—N4	1.945 (3)	C14—C13	1.376 (5)
Cu3—N5	1.954 (3)	C14—C13^i^	1.376 (5)
Cu3—N8	2.004 (3)	C8—C9	1.366 (6)
O1—C15	1.422 (4)	C8—C7	1.370 (6)
Cl1—C5	1.726 (4)	C6—H6	0.9300
Cl4—C12	1.726 (3)	N2—C3	1.333 (5)
Cl2—C2	1.724 (4)	C11—H11	0.9300
Cl3—C8	1.707 (4)	C11—N9	1.331 (5)
Cl5—C14	1.714 (5)	C3—H3	0.9300
C5—C4	1.367 (5)	C9—H9	0.9300
C5—C6	1.374 (5)	C10—H10	0.9300
N3—N4	1.352 (4)	C13—H13	0.9300
N3—C4	1.341 (4)	C7—H7	0.9300
N4—C6	1.332 (4)	C15—H15A	0.9600
N1—C1	1.337 (5)	C15—H15B	0.9600
N1—N2	1.347 (4)	C15—H15C	0.9600
N5—N6	1.341 (4)		
			
O1—Cu1—Cl6	68.85 (8)	N6—N5—Cu3	118.1 (2)
N1—Cu1—O1	88.80 (11)	C7—N5—Cu3	132.8 (3)
N1—Cu1—Cl6	97.90 (10)	C7—N5—N6	108.0 (3)
N1—Cu1—N7	92.60 (13)	C1—C2—Cl2	126.4 (3)
N7—Cu1—O1	174.64 (11)	C3—C2—Cl2	127.5 (3)
N7—Cu1—Cl6	105.83 (10)	C3—C2—C1	106.1 (3)
N6—Cu1—O1	89.16 (11)	N7^i^—N7—Cu1	120.03 (9)
N6—Cu1—Cl6	92.69 (10)	N7^i^—N7—C13	108.5 (2)
N6—Cu1—N1	167.67 (14)	C13—N7—Cu1	131.2 (3)
N6—Cu1—N7	90.55 (13)	C11—C12—Cl4	126.8 (3)
O1—Cu2—Cl6	64.64 (7)	C10—C12—Cl4	126.9 (3)
N3—Cu2—O1	89.11 (11)	C10—C12—C11	106.2 (3)
N3—Cu2—Cl6	90.76 (11)	C5—C4—H4	125.7
N3—Cu2—N2	168.32 (14)	N3—C4—C5	108.7 (3)
N3—Cu2—N9^i^	92.22 (13)	N3—C4—H4	125.7
N2—Cu2—O1	87.86 (11)	C10—N8—Cu3	129.7 (3)
N2—Cu2—Cl6	98.10 (11)	C10—N8—N9	108.0 (3)
N2—Cu2—N9^i^	92.65 (13)	N9—N8—Cu3	120.8 (2)
N9^i^—Cu2—O1	170.36 (11)	N1—C1—C2	108.5 (3)
N9^i^—Cu2—Cl6	105.78 (9)	N1—C1—H1	125.8
O1—Cu3—Cl6	67.41 (7)	C2—C1—H1	125.8
N4—Cu3—O1	88.20 (11)	N5—N6—Cu1	119.1 (2)
N4—Cu3—Cl6	95.32 (11)	C9—N6—Cu1	131.2 (3)
N4—Cu3—N5	171.42 (14)	C9—N6—N5	108.4 (3)
N4—Cu3—N8	92.93 (12)	C13^i^—C14—Cl5	127.2 (2)
N5—Cu3—O1	88.99 (11)	C13—C14—Cl5	127.2 (2)
N5—Cu3—Cl6	91.07 (12)	C13^i^—C14—C13	105.6 (5)
N5—Cu3—N8	90.38 (13)	C9—C8—Cl3	126.8 (3)
N8—Cu3—O1	176.34 (11)	C9—C8—C7	105.5 (3)
N8—Cu3—Cl6	109.00 (9)	C7—C8—Cl3	127.7 (3)
Cu1—O1—Cu2	102.70 (10)	C5—C6—H6	125.5
Cu1—O1—Cu3	103.49 (10)	N4—C6—C5	108.9 (3)
Cu2—O1—Cu3	105.62 (10)	N4—C6—H6	125.5
C15—O1—Cu1	114.7 (2)	N1—N2—Cu2	115.6 (2)
C15—O1—Cu2	113.9 (2)	C3—N2—Cu2	134.8 (3)
C15—O1—Cu3	115.0 (2)	C3—N2—N1	108.5 (3)
Cu1^i^—Cl6—Cu1	87.60 (10)	C12—C11—H11	125.6
Cu1^i^—Cl6—Cu2	138.95 (6)	N9—C11—C12	108.9 (3)
Cu1—Cl6—Cu2^i^	138.95 (6)	N9—C11—H11	125.6
Cu1^i^—Cl6—Cu2^i^	78.02 (2)	C2—C3—H3	125.6
Cu1—Cl6—Cu2	78.02 (2)	N2—C3—C2	108.8 (3)
Cu1^i^—Cl6—Cu3^i^	81.39 (2)	N2—C3—H3	125.6
Cu1—Cl6—Cu3	81.39 (2)	N6—C9—C8	109.0 (3)
Cu1—Cl6—Cu3^i^	136.85 (6)	N6—C9—H9	125.5
Cu1^i^—Cl6—Cu3	136.85 (6)	C8—C9—H9	125.5
Cu2^i^—Cl6—Cu2	135.50 (12)	C12—C10—H10	125.7
Cu3^i^—Cl6—Cu2	83.43 (5)	N8—C10—C12	108.5 (3)
Cu3^i^—Cl6—Cu2^i^	79.05 (4)	N8—C10—H10	125.7
Cu3—Cl6—Cu2^i^	83.43 (5)	N7—C13—C14	108.7 (4)
Cu3—Cl6—Cu2	79.05 (4)	N7—C13—H13	125.7
Cu3^i^—Cl6—Cu3	132.63 (12)	C14—C13—H13	125.7
C4—C5—Cl1	126.5 (3)	N5—C7—C8	109.0 (4)
C4—C5—C6	106.0 (3)	N5—C7—H7	125.5
C6—C5—Cl1	127.6 (3)	C8—C7—H7	125.5
N4—N3—Cu2	120.5 (2)	N8—N9—Cu2^i^	120.5 (2)
C4—N3—Cu2	131.1 (3)	C11—N9—Cu2^i^	130.8 (3)
C4—N3—N4	108.3 (3)	C11—N9—N8	108.4 (3)
N3—N4—Cu3	118.1 (2)	O1—C15—H15A	109.5
C6—N4—Cu3	133.5 (3)	O1—C15—H15B	109.5
C6—N4—N3	108.2 (3)	O1—C15—H15C	109.5
C1—N1—Cu1	131.9 (3)	H15A—C15—H15B	109.5
C1—N1—N2	108.1 (3)	H15A—C15—H15C	109.5
N2—N1—Cu1	120.0 (2)	H15B—C15—H15C	109.5
			
Cu1—N1—C1—C2	−176.5 (3)	N4—N3—C4—C5	1.0 (4)
Cu1—N1—N2—Cu2	−12.9 (4)	N1—N2—C3—C2	0.1 (5)
Cu1—N1—N2—C3	177.1 (3)	N5—N6—C9—C8	0.1 (5)
Cu1—N7—C13—C14	174.5 (2)	N7^i^—N7—C13—C14	0.6 (5)
Cu1—N6—C9—C8	−166.4 (3)	C12—C11—N9—Cu2^i^	173.2 (3)
Cu2—N3—N4—Cu3	−7.5 (4)	C12—C11—N9—N8	0.0 (4)
Cu2—N3—N4—C6	177.2 (2)	C4—C5—C6—N4	0.7 (4)
Cu2—N3—C4—C5	−176.4 (3)	C4—N3—N4—Cu3	174.8 (2)
Cu2—N2—C3—C2	−167.2 (3)	C4—N3—N4—C6	−0.5 (4)
Cu3—N4—C6—C5	−174.5 (3)	C1—N1—N2—Cu2	169.6 (3)
Cu3—N5—N6—Cu1	−1.4 (4)	C1—N1—N2—C3	−0.4 (5)
Cu3—N5—N6—C9	−169.8 (3)	C1—C2—C3—N2	0.3 (5)
Cu3—N5—C7—C8	167.7 (3)	N6—N5—C7—C8	0.4 (5)
Cu3—N8—C10—C12	−165.8 (3)	C6—C5—C4—N3	−1.0 (4)
Cu3—N8—N9—Cu2^i^	−6.7 (4)	N2—N1—C1—C2	0.6 (5)
Cu3—N8—N9—C11	167.3 (3)	C11—C12—C10—N8	0.1 (5)
Cl1—C5—C4—N3	178.9 (3)	C3—C2—C1—N1	−0.6 (5)
Cl1—C5—C6—N4	−179.2 (3)	C9—C8—C7—N5	−0.4 (6)
Cl4—C12—C11—N9	−176.8 (3)	C10—C12—C11—N9	0.0 (5)
Cl4—C12—C10—N8	176.9 (3)	C10—N8—N9—Cu2^i^	−173.9 (3)
Cl2—C2—C1—N1	−179.4 (3)	C10—N8—N9—C11	0.1 (4)
Cl2—C2—C3—N2	179.1 (3)	C13^i^—C14—C13—N7	−0.24 (19)
Cl3—C8—C9—N6	177.6 (3)	C7—N5—N6—Cu1	168.1 (3)
Cl3—C8—C7—N5	−177.8 (4)	C7—N5—N6—C9	−0.3 (5)
Cl5—C14—C13—N7	179.76 (19)	C7—C8—C9—N6	0.2 (6)
N3—N4—C6—C5	−0.1 (4)	N9—N8—C10—C12	−0.1 (4)

Symmetry code: (i) −*x*, *y*, −*z*+1/2.

### Hydrogen-bond geometry (Å, º)

**Table:**

*D*—H···*A*	*D*—H	H···*A*	*D*···*A*	*D*—H···*A*
C1—H1···Cl4^ii^	0.93	2.75	3.586 (4)	149
C6—H6···Cl3^iii^	0.93	2.81	3.466 (4)	129
C15—H15*A*···Cl3^iv^	0.96	2.82	3.651 (4)	146

Symmetry codes: (ii) *x*−1/2, *y*+1/2, −*z*+1/2; (iii) −*x*+1/2, *y*−1/2, *z*; (iv) −*x*+1/2, −*y*+1/2, *z*+1/2.
